# Cysteine-silver-gold Nanocomposite as potential stable green corrosion inhibitor for mild steel under acidic condition

**DOI:** 10.1038/s41598-019-57181-5

**Published:** 2020-01-14

**Authors:** Megha Basik, Mohammad Mobin, Mohd Shoeb

**Affiliations:** Corrosion Research Laboratory, Department of Applied Chemistry, Faculty of Engineering and Technology, Aligarh Muslim University, Aligarh, 202002 India

**Keywords:** Chemistry, Materials science

## Abstract

Cysteine based silver-gold nanocomposite (Cys/Ag-Au NCz) was synthesized, this was followed by its characterization using Fourier-transform infrared spectroscopy (FTIR), X-ray diffraction (XRD), Ultraviolet-visible spectroscopy (UV-Vis), Scanning electron microscopy (SEM), Energy-dispersive X-ray spectroscopy (EDAX), Thermogravimetric analysis (TGA) and Transmission electron microscopy (TEM). Cys/Ag-Au NCz was studied as novel green corrosion inhibitor for mild steel in 1M HCl solution at varying concentration and temperature using gravimetric, Potentiodynamic polarization (PDP), Electrochemical impedance spectroscopy (EIS), SEM, EDAX and FTIR. Weight loss, PDP and EIS studies confirmed Cys/Ag-Au NCz as efficient corrosion inhibitor at moderately low concentration. The maximum inhibition efficiency of 96 % was observed at 303 K at 300 ppm. Cys/Ag-Au NCz acted by affecting both anodic and cathodic processes and its adsorption on steel surface followed the Langmuir adsorption isotherm. EIS data displayed the existence of protective film at mild steel/solution interface in Cys/Ag-Au NCz inhibited system. SEM micrograph in presence of Cys/Ag-Au NCz inhibited acid solution displayed better morphology as compared to blank solution. The UV-Vis and FTIR data indicates good interaction between the Cys/Ag-Au NCz and steel surface.

## Introduction

The appropriate mechanical properties of mild steel makes it an important raw material to find application in various industries like construction, petroleum, power and water generation, etc. But due to its reactive property and thermodynamic instability it is susceptible to corrosion. Hydrochloric acid is widely used in industries for oxides removal, pickling, industrial cleaning etc., which causes huge metallic losses and damage to system components. The use of corrosion inhibitors is the most effective and economic method to prevent the metal against corrosion and increase its durability^1^. Synthetic organic inhibitors have been found to be effective in terms of reducing steel dissolution but due to environmental threat their usage is limited. Thus current researches have been focused on using cheap, non-toxic and biodegradable corrosion inhibitors^2^. Presence of heteroatom’s and pi-electrons in the molecular structure of an inhibitor plays a key role in determination of its inhibitive performance. Amino acids qualifies as one of the such compounds and are reported to serve as effective inhibitor against corrosion in various aggressive medium^3^. However, use of single amino acids generally needs high amount of inhibitor usage. The idea of synergism had been used since decades and found to be effective in reducing inhibitor concentration and consequently favoring its economic viability and practical applicability. Alkali metal halides, metal ions and surfactants are some of additives which have been used as synergistic agent to several natural and synthetic organic compounds^4,5^. Another alternative to decrease the inhibitor dosage is the infusion of certain inorganic substance to make composites which reduce the particle size and hereby increase the surface coverage and thus protect metal against corrosion. Silver and gold have been widely used for composite formation when introduced in small amount to certain compounds^6^. Looking at the potentiality of such composites we have synthesized a new cysteine/Ag-Au nanocomposite and examined its potentiality in minimizing corrosion of mild steel in HCl solution. The active sites of cysteine are amine group, carboxylic group and sulfur group. These groups have electron rich atoms such as nitrogen, oxygen and sulfur. They can easily donate electron to 5d and 6s orbitals of Au and 4d and 5s orbitals of Ag via lone pair interaction. Ag and Au also act as proton acceptor and form nonconventional hydrogen bonds with amine and hydroxyl groups. The interaction between amino acid and silver-gold is either monodentate or bidentate. Thus the synthesized compound is expected to form large complex (cluster) type structure which may cover large surface area of metals and provide more effective inhibition^7^.

## Experimental

### Materials and chemicals

Cysteine, HAuCl_4_ (Tetrachloroauric acid), AgNO_3_ (Silver Nitrate) and CTAB (Cetyl Trimethyl Ammonium Bromide) were purchased from Sigma Aldrich. Pomegranate was purchased from local market. A1020 steel of chemical composition listed in Table 1 was used for corrosion study. The rectangular shape coupons of 2.5 × 2.0 × 0.1 cm (dimension) were used for surface analysis and weight loss analysis while circular coupons of dimension 1 cm^2^ in diameter were used for EIS and PDP studies.

**Table 1:** Chemical composition of mild steel.

Elements	% Composition
C	0.06841
Mn	0.03939
S	0.00080
P	0.02197
Cr	0.04561
Mo	0.06743
Al	0.01539
V	0.03347
Fe	Remaining

### Extraction of pomegranate extract

The pomegranate fruit, obtained from a local market, was successively cleaned with tap water and distilled water to eliminate dust and undesirable observable contaminants. Pomegranate seed was detached from the fruit, ground and extract was stored in a beaker (250 mL). For this, 50 mL double distilled water was introduced and the mixture was boiled for 5min. Following that, the liquid was filtered and kept at 4 ºC and named PFE^8^.

### Synthesis of Ag-Au nanocomposite

To synthesize Ag-Au NCz, 10 mM CTAB, 5.0 mM AgNO_3_ (3mL) and 5.0 mM HAuCl_4_ were added to 50.0 mL distilled water^9^. 20 mL of PFE was introduced into this solution drop wise and exposed inside a conventional microwave oven (Samsung Electronics, 300 W) for complete reduction for 3 min. The color of the solution altered to brownish-red, which revealed the formation of Ag-Au NCz. The Ag-Au NCz was harvested, centrifuged and kept in vacuum desiccators.

### Synthesis of cysteine functionalized Ag-Au nanocomposite

Synthesized Ag-Au NCz was functionalized with cysteine. An amount of synthesized Ag-Au NCz (100 mg) powder was consistently dispersed in 50 mL of double distilled water with ultrasonication. Subsequently, the solution formulated with 1 M cysteine and 1 mM NH_3_ was incorporated. Eventually, the resultant solution was intensely stirred at room temperature for 72 h. The resulting precipitate was rinsed six times with a solution of ethanol-water (1:1) and dried at 313K for 12 h^10^.

### Preparation of test sample and test solution

#### Preparation of test sample

Before starting the corrosion inhibition experiments the rectangular and circular shaped coupons were polished properly to get a smooth surface which is free from pits. The polishing of these coupons was achieved from different grades (320–1200) of emery papers, this was followed by washing with double distilled water, degreasing with acetone and finally subjecting to warm air.

#### Preparation of test solution

37% Analytical grade HCl was used for preparing 1 M HCl solution. 300 ppm stock solution was prepared by adding Cys/Ag-Au NCz in 1 M HCl. Solution of distinct concentration (30–200 ppm) was prepared by diluting the stock solution.

### Characterization of Cys/ Ag-Au NCz

#### FT-IR studies

Perkin Elmer Spectrometer with spectral resolution of 0.5 cm^−1^ is used for analysis in range of 400–4000 cm^−1^ frequency. KBr pellet was prepared in the ration of 1:100 and then spectra was recorded. The FT-IR was conducted to evaluated the different functional groups present in the synthesized compound.

#### XRD analysis

Bruker D8 Advance X-ray powder diffractometer was used to record the XRD pattern of Cys/Ag-Au NCz. The sample was radiated with Cu Kα at 35 KV and a current of 10 mA. The diffracted intensities were recorded over the range of 2θ = 5–85°.

#### UV-Vis analysis

Perkin Elmer spectrophotometer (Lambda 25) with UV Winlab Data Processor and Viewer was used to identify the complex formation.

#### Scanning electron microscopy and EDAX

JSM-6510 LV scanning electron microscope (JEOL, Japan) model equipped with EDAX (INCA, Oxford instrument) attachment was used for surface morphological studies and compositional information.

#### Transmission electron microscopy

For TEM analysis, a copper grid loaded with sample was air dried and kept under dark. JEOL 100/120 kV transmission electron microscope was used at an accelerating voltage of 80 kV.

#### Thermogravimetric analysis

The thermal stability of the synthesized nanocomposite was investigated by thermogravimetric analysis (Sieco SII, SSC5100, Instrument). The heating rate of 10 °C min^−1^ was used under nitrogen atmosphere.

### Corrosion inhibition tests

#### Weight loss measurement

The prepared coupons of rectangular shape were weighed before immersion in test solution. The studies were performed on triplicate set of coupons at each concentration for a duration of 6 h as per relevant ASTM designation. The test was conducted at 303 K, 313 K, 323 K and 333 K. After completion of test run the coupons were removed from test solution, corrosion product removed in accordance with ASTM designation, washed with distilled water, dried, reweighed and difference in weight was used to measure the corrosion rate and inhibition efficiency (%I.E).

1 $${\rm{CR}}=\frac{\Delta {\rm{W}}}{{\rm{AT}}}$$

2 $$ \% {\rm{IE}}=\frac{{{\rm{CR}}}_{0}-{{\rm{CR}}}_{{\rm{i}}}}{{{\rm{CR}}}_{0}}\times 100$$

where, CR, ΔW, A, T, CR_o_ and CR_i_ are coupon corrosion rate (mg cm^−2^ h^−1^), loss in weight (mg), sample area (cm^2^), immersion time (hr) and corrosion rates with or without inhibitor.

#### Electrochemical analysis

**Figure 1:**
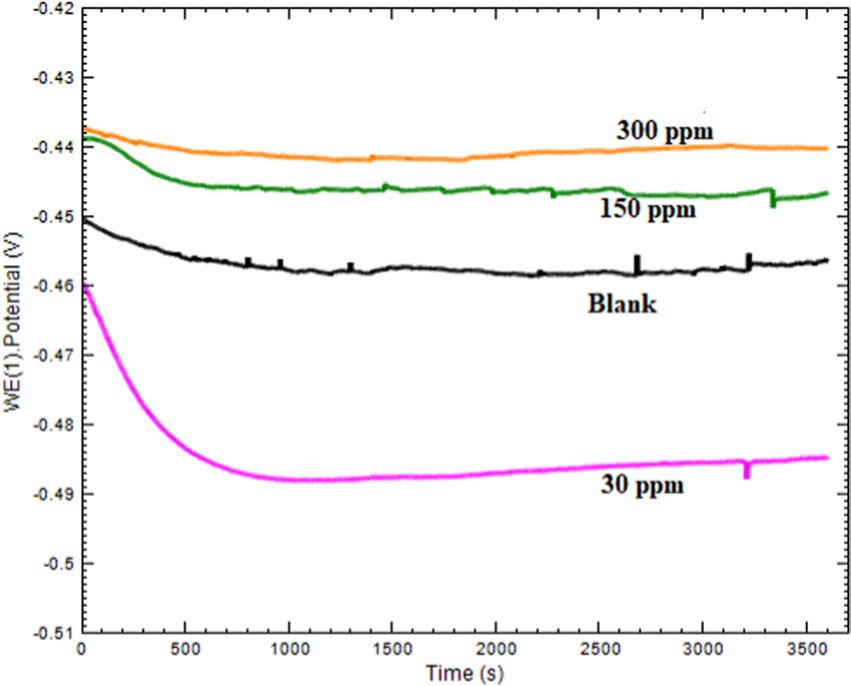
Eocp-time curves for mild steel in 1 M HCl solution without and with different concentrations of Cysteine/Ag-Au nanocomposite.at 303 K.

Electrochemical tests (Instrumental test) was conducted by using circular coupons. The test was conducted in 1 L corrosion cell with three electrode system in which working electrode is mild steel sample, reference electrode is Ag/AgCl with KCl and counter electrode is platinum wire. IR drop was minimized by inserting capillary known as Luggin Haber in parallel to working electrode. Autolab Metrohm 128 N instrument was used for conducting tests. Before EIS and PDP measurements, the OCP (open circuit potential) was conducted to stabilize the system and attain the steady state, which was achieved after approximately 60 min (Fig. 1). For EIS measurements the used frequency range is 10^–2^ Hz to 10^5^ Hz with 10 mV peak to peak perturbation. The PDP measurements was conducted in the potential range of −250 to +250 mV with rate of scan being 0.0001 V/s.

## Results and Discussion

### Characterization of Cys/Ag-Au NCz

#### FT-IR analysis

**Figure 2:**
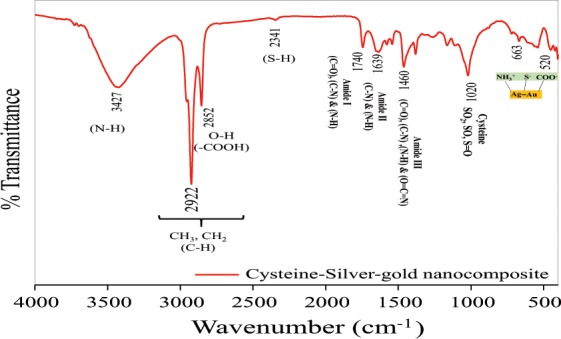
FT-IR spectra of Cysteine/Ag-Au nanocomposite.

The FTIR spectrum of Cys/Ag-Au NCz is shown in Fig. 2. The peak at 3427 cm^−1^ is assigned to N–H stretching vibration of the NH_3_^+^ group in the cysteine functionalized Ag-Au nanocomposite. The peak at 2341 cm^−1^ is assigned to S–H vibration in the thiol group with minimum intensity group and may be attributed to the deprotonation of the thiol group in the cysteine molecule that is the pre-requisite for the formation of Au-Ag–S bonding with cysteine. Moreover, peak at 1740 cm^−1^, 1639 cm^−1^ and 1460 cm^−1^ belongs to amide I [(C=O), (C-N) and (N-H)], amide II [(C-N) and (N-H] and amide III [(C=O), (C-N), (N-H) and (O=C=N)], respectively. Furthermore, the peak at 1020 cm^−1^ is attributed to cysteine S-O vibration SO_2_, SO, S=O and lower frequency at 663 cm^−1^ and 520 cm^−1^ suggests that there exists a bonding interaction between the Au-Ag and either or all the three S^−^, COO^−^ and NH_3_^+^ groups of the functionalized cysteine molecules^11^.

#### XRD analysis

**Figure 3:**
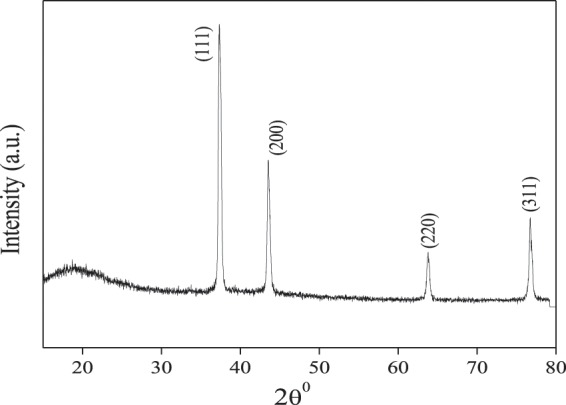
XRD pattern of Cysteine/Ag-Au nanocomposite.

XRD pattern of cysteine functionalized Ag-Au nanocomposite (Fig. 3) clearly shows the diffraction peak at 37.48°, 43.35°, 63.86° and 76.89° matching to the (111), (200), (220), and (311) miller indices of polycrystal of Ag-Au nanocomposite. The interatomic distances to the silver nanoparticles and gold nanoparticles are extremely closer to be differentiated with conventional XRD analysis^12^. Furthermore, a broad hump is observed at 20^0^ which might be due to successful functionalization of cysteine. The average crystallite size (D) of the nanocomposite was determined following the Debye-Scherer formula^13,14^:

3 $${\rm{D}}=\frac{{\rm{k}}{\rm{\lambda }}}{{\rm{\beta }}\,\cos \,{\rm{\theta }}}$$

where k = 0.9 is the shape factor, λ is the X-ray wavelength of Cu Kα radiation (1.54 Å), θ is the Bragg diffraction angle, and β is the full width at half maximum height (FWHM) of the (111) plane diffraction peak. The determined average crystalline size was observed to be 22 nm of Cys/Ag-Au NCz. From the XRD it was confirmed that the Cys/Ag-Au NCz are in the uniform phase, and the functionalization of the cysteine on Ag-Au nanoparticles was achieved properly.

#### UV-Vis analysis

The complexation between Cys/Ag-Au NCz and Fe was confirmed by UV-Vis spectroscopy. The spectra of Cys/Ag-Au NCz containing 1 M HCl before and after immersion of mild steel coupon at 303K for duration of 6 h are shown in Fig. S1. The spectrum of solution containing Cys/Ag-Au NCz before immersion of mild steel coupon shows two characteristic peaks at 208.7 nm and 248.2 nm with absorbance of 1.73 and 0.77 while the solution containing Cys/Ag-Au NCz after immersion of mild steel coupon shows visible shift in wavelength to 225.5 nm and 253.0 nm with hike in absorbance of 3.61 and 3.16. The increase in the peaks absorbance indicate good complex formation between metal and inhibitor in acidic medium.

#### SEM and EDAX analysis

**Figure 4:**
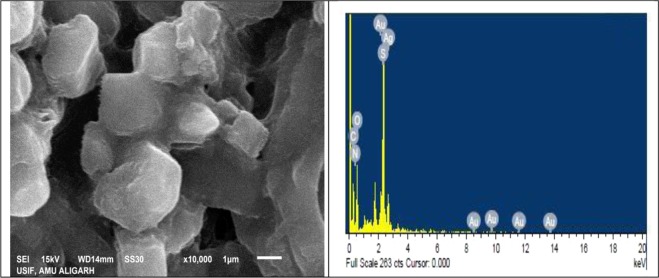
SEM & EDAX images of Cysteine/Ag-Au nanocomposite.

**Figure 5:**
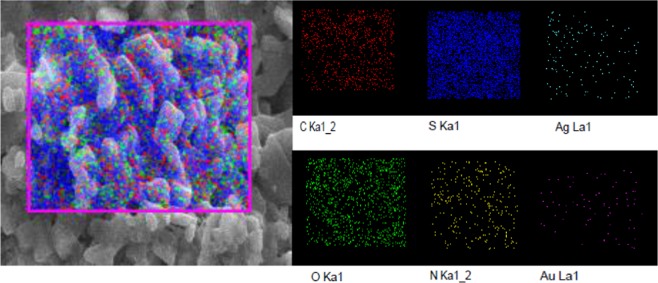
EDAX mapping of Cysteine/Ag-Au nanocomposite.

SEM analysis has been conducted for synthesized Cys/Ag-Au NCz to know the surface morphology of the compound (Fig. 4a). The spherical amorphous shape of silver-gold was obtained with hazy surroundings of irregular shaped particles of functionalized cysteine. The elemental analysis and percent of incorporation can be judged by EDAX (Fig. 4b) and mapping (Fig. 5). EDAX spectrum shows visible peaks of C, O, N, S, Ag and Au which clearly reveals the inclusion of Ag-Au nanocomposite into the cysteine. Mapping provide the easy illustration of different amount of elements present in Cys/Ag-Ag NCz. The red color dots stands for carbon, green color for oxygen, blue color for sulphur, yellow color for nitrogen, sea blue color for silver and pink color for gold, respectively.

#### TEM analysis

**Figure 6:**
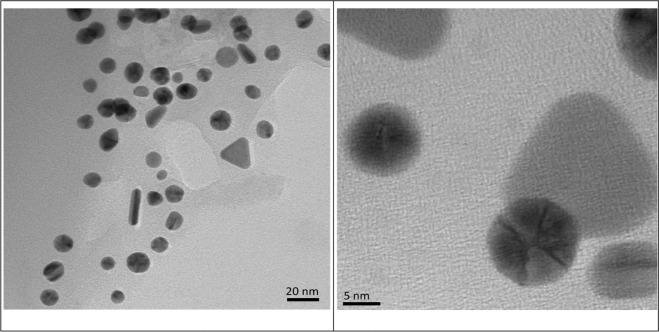
TEM micrograph of Cysteine/Ag-Au nanocomposite.

The particle size investigation suggests that the synthesized nanoparticles have average particle size of about 17–20 nm (Fig. 6). Spherical nanoparticles (possibly silver nanoparticle) of around 17 nm diameter and trianglular nanoparticles (possibly gold nanoparticle) of around 20 nm diameter could be distinctly observed in the TEM photomicrographs. The Ag-Au nanocomposite with cysteine functionalized on the exterior surface was also seen into the TEM images. A fine covering layer of cysteine is existing around the surface of the Ag-Au nanocomposite.

#### TGA analysis

**Figure 7:**
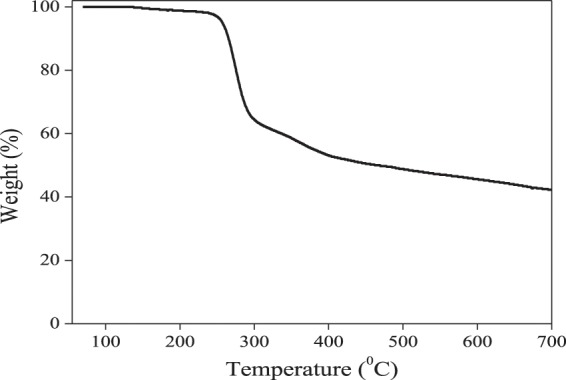
Thermogravimetric curve for Cysteine/Ag-Au nanocomposite.

Thermogravimetric analysis was undertaken to evaluate the thermal stability of the cysteine functionalize Ag-Au nanocomposite (Fig. 7). The thermograms revealed significant weight loss at the studied temperature range. The weight loss at the transition from room temperature to 196 °C is most likely to the evaporation of physically adsorbed H_2_O molecules. The weight loss from 200 to 430 °C might be assigned to the decomposition of cysteine molecules existing in the nanocomposite. Additional weight loss between 500 and 700°C may be due to the combustion of passive organic compounds. It is plausible to consider that the higher residual weights of the synthesized nanocomposite was attributable to the functionalized silver-gold nanoparticles because the metal nanoparticles are not anticipated to disintegrate at the subjected temperatures.

## Corrosion Studies

### Weight loss experiment

#### Effect of inhibitor concentration and temperature

Corrosion rate and inhibition efficiency was calculated for Cys/Ag-Au NCz at different concentrations and temperatures ranging from 303 to 333K. The obtained parameters are listed in Table 2. It is clearly seen that both concentration and temperature are affecting the corrosion rate. The maximum % IE obtained was 95 % at 300 ppm at 303 K. As the dosing of Cys/Ag-Au NCz in acid increases % IE also increases but after approaching the optimum concentration (300 ppm) the effectiveness of inhibitor starts diminishing probably due to the back flow of adsorbed molecules in the solution. On the other hand on raising the temperature from 303 to 333 K the % IE starts declining which is due to the weakening of Vander Waal’s interaction build between inhibitor molecules and metal surface i.e., inhibitor is physically adsorbed on metal’s surface^15^.

**Table 2:** Corrosion parameters for mild steel in 1 M HCl in absence and presence of the Cys/Ag-Au NCz from weight loss measurements at different temperatures.

	Temperature															
	303 K				313 K				323 K				333 K			
Inhibitor conc. (ppm)	CR (mgcm^−2^h^−1^)	SD	θ	% IE	CR (mgcm^−2^h^−1^)	SD	θ	% IE	CR (mgcm^−2^h^−1^)	SD	θ	% IE	CR (mgcm^−2^h^−1^)	SD	θ	% IE
0	0.155	0.002			0.441	0.031			2.944	0.006			6.772	0.095		
50	0.055	0.005	0.64	64.59	0.176	0.006	0.60	60.05	1.262	0.003	0.57	57.12	3.588	0.075	0.47	47.02
100	0.038	0.002	0.75	75.74	0.136	0.011	0.69	69.17	1.079	0.048	0.63	63.33	3.171	0.085	0.53	53.18
150	0.028	0.002	0.82	82.29	0.110	0.005	0.75	75.17	0.942	0.022	0.68	68.01	2.830	0.025	0.58	58.20
200	0.012	0.002	0.92	92.13	0.060	0.005	0.86	86.37	0.669	0.060	0.77	77.25	2.202	0.075	0.67	67.48
300	0.008	0.002	0.95	95.08	0.045	0.005	0.89	89.84	0.549	0.094	0.81	81.34	1.924	0.026	0.71	71.59

#### Effect of time

**Figure 8:**
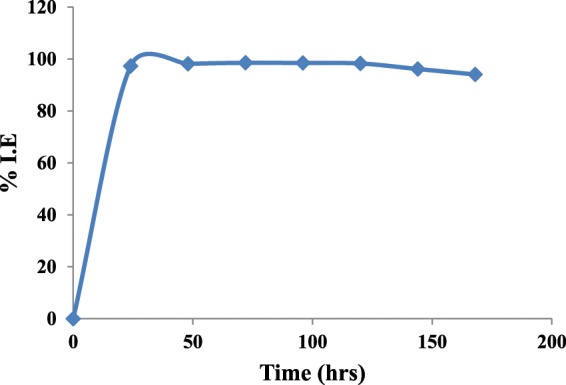
Plot of %I.E vs. immersion time for mild steel in 1 M HCl with 300 ppm concentration of Cysteine/Ag-Au nanocomposite at 303 K.

To establish the stability of Cys/Ag-Au NCz in HCl over long exposure period the weight loss measurements at optimum concentration of Cys/Ag-Au NCz at 303K was conducted for an expanded period of 168 hrs. The plot of day’s stability is shown in Fig. 8. It is indicated from the graph that % I.E is improved with passage of time till certain period which is due to the increase in the strength of adsorbed film with time. After certain period of time % I.E become nearly constant which confirmed the stability of Cys/Ag-Au NCz in HCl solution over long period of time.

#### Thermodynamic and activation parameters

Arrhenius equation was used for calculating various activation parameters at temperature range of 303–333 K.

4 $${\rm{logCR}}=\frac{-{\rm{Ea}}}{2.303{\rm{RT}}}+{\rm{logA}}$$

where Ea is energy of activation, R is gas constant (8.314 J K^−1^ mol^−1^), T is temperature in K and A is pre-exponential factor. The logarithm of CR against 1/T (Fig. S2) had been constructed in absence and presence of various concentration of inhibitor and value of Ea from slope (−Ea/R) of Arrhenius plot was calculated. Pre-exponential factor (A) was calculated by intercept of plot. All the parameters are mentioned in Table 3. High value of Ea was obtained in inhibited medium as compared to uninhibited medium, which suggests physical adsorption^16^. Both Ea and A influence the process of corrosion. As the value of Ea increases with increasing concentration of inhibitor, the CR starts decreasing. On the other hand the value of A increases with increasing the concentration of inhibitor, which suggests enhancement in CR. These both observations are contradictory to each other hence the deciding factor may invariably vary according to the surroundings. So here in present study the deciding factor is Ea which affects the rate of corrosion.

**Table 3:** Activation parameters for mild steel in 1 M HCl in the absence and presence of different concentrations of the Cys/Ag-Au NCz.

Inhibitor concentration (ppm)	Pre-exponential factor (A) (g m^−2^ h^−1^)	Ea (kJ mol^−1^)	ΔH* (kJ mol^−1^)	ΔS* (J mol^−1^K^−1^)
0	86789829	110.96	108.32	96.23
30	3.46 × 10^8^	121.64	118.99	122.73
50	3.98 × 10^8^	121.95	119.31	125.38
100	1.04 × 10^9^	128.95	126.31	143.84
150	2.48 × 10^9^	134.73	132.09	160.42
200	2.92 × 10^10^	151.05	148.41	207.64
300	1.19 × 10^11^	160.34	157.70	234.61

Activation enthalpy (ΔH*) and activation entropy (ΔS*) was computed by using Eyring transition state equation^17^. Value of ΔH* and ΔS* was obtained from the slope and intercept of log CR/T vs. 1/T (Fig. S3). All the obtained parameters are mentioned in Table 3. The value of ΔH* is positive which reveals the endothermic process of steel dissolution in absence and presence of inhibitor. Both Ea and ΔH* increases on increasing concentration of inhibitor suggesting the enhancement in barrier of energy for mild steel corrosion which leads to obstruct the corrosion reaction at mild steel surface. The value of ΔS* of the system in presence of Cys/Ag-Au NCz is higher than in its absence and progressively increases on increasing its concentration. This is suggestive of disordering of the inhibitor containing systems which took place while going from reactants to activated complex. The adsorption of Cys/Ag-Au NCz molecules from HCl solution can be regarded as quasi-substitutional process between NCz molecules in the aqueous phase and water molecules already adsorbed on the mild steel surface. In such situation, the adsorption of Cys/Ag-Au NCz molecules is accompanied by desorption of water molecules from mild steel surface. ΔS* values obtained are the algebraic sum of adsorption of inhibitor molecules and desorption of water molecules. Therefore, the gain in entropy is attributed to the increase in the solvent entropy and to more positive water desorption entropy^18,19^.

#### Adsorption isotherms

The mode of adsorption and the illustration regarding the interaction of inhibitor molecules on the metal’s surface can be judged by fitting the adsorption isotherms. Various isotherms had been studied and the best fitting is obtained for Langmuir isotherm with value of R^2^ close to unity.

5 $$\frac{{\rm{C}}}{{\rm{\theta }}}=\frac{1}{{{\rm{K}}}_{{\rm{ads}}}}+{\rm{C}}$$

where θ is the surface coverage by inhibitor molecule, C is the concentration and K_ads_ is adsorption constant. Fig. S4 shows the Langmuir plot (C/θ vs C) for varying concentration of Cys/Ag-Au NCz at temperature range of 303–333 K. The value of K_ads_ was obtained by intercept of Langmuir plot. The value of ΔG_ads_ (free energy of adsorption) can be calculated as:

6 $${\Delta {\rm{G}}}_{{\rm{ads}}}=-{\rm{RTln}}(1\times {10}^{6}{{\rm{K}}}_{{\rm{ads}}})$$

where 1 × 10^6^ is the concentration of water in ppm. The factor 55.5 (mol L^−1^) is replaced by 1 × 10^6^ to meets the unit of K_ads_, T is the temperature in K and R is the universal gas constant^20^. Obtained adsorption values are mentioned in Table 4. The spontaneity of the process can be judged by the negative value of ΔG_ads_. It is clearly seen from Table 4 that value of ΔG_ads_ is negative suggesting that formed layer on mild steel surface is stable and process of adsorption is feasible. Enthalpy of adsorption (ΔH_ads_) and entropy of adsorption (ΔS_ads_) can be obtained by plotting the graph between ln K_ads_ and 1/T (Fig. S5). From the slope and intercept of the plot values of ΔH_ads_ and ΔS_ads_ can be calculated using following equation:

7 $${{\rm{lnK}}}_{{\rm{ads}}}=-\frac{{\Delta {\rm{H}}}_{{\rm{ads}}}}{{\rm{RT}}}+\frac{{\Delta {\rm{S}}}_{{\rm{ads}}}}{{\rm{R}}}-\,\mathrm{ln}(1\times {10}^{6})$$

The value of ΔH_ads_ is negative which signifies the exothermic process of inhibitor adsorption. In exothermic process, for phyisorption the value of ΔH_ads_ is less than −40 kJ mol^−1^ and for chemisorption the value of ΔH_ads_ is greater than −40 kJ mol^−1^ and approaches to −100 kJ mol^−1^ ^21^. The obtained value of ΔH_ads_ for Cys/Ag-Au NCz was less than −40 kJ mol^−1^ revealing physical adsorption process of inhibitor. The value of ΔS_ads_ was positive which was due to desorption of more water molecules by inhibitor molecules.

**Table 4:** The thermodynamic parameters of adsorption of the inhibitor at different concentrations for mild steel in 1 M HCl solution.

Temperature (K)	Method	R^2^	K_ads_	ΔG_ads_ (kJ/mol)	ΔH_ads_ (kJ/mol)	ΔS_ads_ (kJ/K.mol)
303	WL	0.99	0.033	−26.21	−9.495	0.055
313	WL	0.99	0.028	−26.65	−9.495	0.055
323	WL	0.99	0.026	−27.32	−9.495	0.055
333	WL	0.98	0.023	−27.82	−9.495	0.055

#### Potentiodynamic polarization measurements

**Figure 9:**
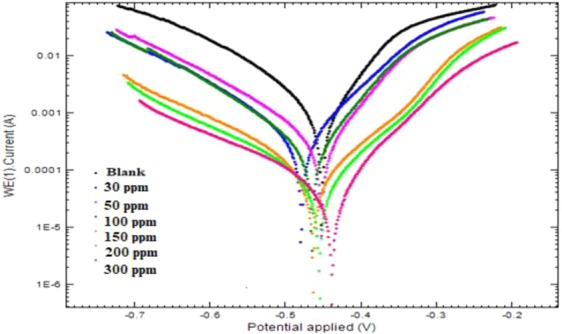
Tafel plots of mild steel in 1 M HCl in the absence and presence of different concentrations of Cysteine/Ag-Au nanocomposite at 303 K.

PDP curves of mild steel immersed in blank medium and inhibited medium are shown in Fig. 9. PDP curve helps us to evaluate the kinetics of cathodic and anodic corrosion reactions. Polarization parameters like E_corr_, I_corr_, β_a_, β_c_ and CR are obtained by extrapolating the cathodic and anodic branches.

8 $$ \% {\rm{I}}{\rm{.E}}=\frac{{{\rm{I}}}_{{\rm{corr}}}^{0}-{{\rm{I}}}_{{\rm{corr}}}}{{{\rm{I}}}_{{\rm{corr}}}^{0}}\times 100$$

where I^0^_corr_ is the corrosion current density in blank solution whereas I_corr_ is the corrosion current density in inhibited solution. All the potentiodynamic parameters are mentioned in Table 5. The mode of inhibition was determined by the E_corr_ value. If the shift in E_corr_ is greater than 85 mV then it can either be anodic or cathodic type of inhibitor whereas if the displacement in E_corr_ is less than 85 mV it is considered to be mixed type of inhibitor. Here, in present study the magnitude of shift in E_corr_ value lies below 85 mV so it behave as mixed type of inhibitor. The value of I_corr_ decreases and consequently %I.E increases on increasing the concentration of inhibitor suggesting better protection and better inhibitive effect against corrosion. The value of both β_a_ and β_c_ changes on adding the inhibitor suggesting that both anodic dissolution of mild steel and cathodic hydrogen evolution process get affected.

**Table 5:** Potentiodynamic polarization results for mild steel in 1 M HCl without and with different concentrations of the Cys/Ag-Au NCz at 303 K.

Inhibitor concentration (ppm)	E_corr_ (mv)	I_corr_ (A/cm^2^)	βa (mV/dec)	βc (mV/dec)	% I.E
0	−452.65	7.98 × 10^−4^	85.10	67.70	
30	−479.80	3.57 × 10^−4^	121.11	86.11	55.25
50	−453.03	2.61 × 10^−4^	107.87	67.89	67.28
100	−465.58	2.24 × 10^−4^	111.83	71.88	71.96
150	−448.76	8.86 × 10^−5^	122.28	77.64	88.90
200	−455.75	6.66 × 10^−5^	137.63	101.44	91.66
300	−439.24	4.78 × 10^−5^	158.89	81.85	94.01

#### Electrochemical impedance spectroscopy measurements

**Figure 10:**
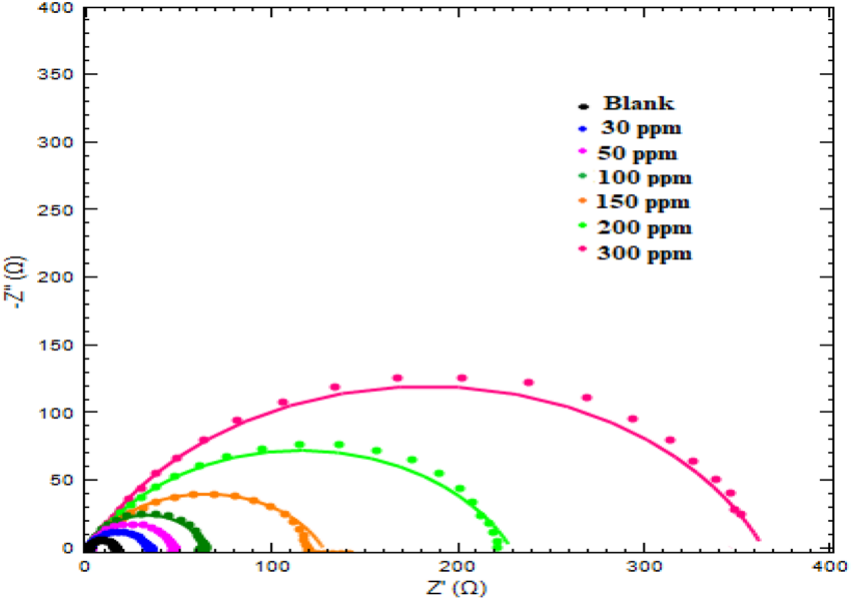
Nyquist plot of Cysteine/Ag-Au nanocomposite at 303 K.

The interfacial kinetics between metal and the corrosive solution was well studied by EIS. Impedance diagrams (Nyquist and Bode), which illustrates the surface properties and kinetics of electrode reactions in 1 M HCl medium in absence and presence of different concentration of inhibitor are depicted in Figs. 10 and S6. Proper impedance data was obtained by appropriately fitting the equivalent circuit to impedance spectra. The circuit comprises to three elements (R_s_, R_ct_ and C_dl_). R_ct_ (charge transfer resistance) and C_dl_ (double layer capacitance) are connected in parallel with R_s_ (solution resistance). The %I.E was calculated from R_ct_ and R^0^_ct_, the charge transfer resistance in blank and inhibited medium respectively, by the following relationship:

9 $$ \% {\rm{I}}{\rm{.E}}=\frac{{{\rm{R}}}_{{\rm{ct}}}-{{\rm{R}}}_{{\rm{ct}}}^{0}}{{{\rm{R}}}_{{\rm{ct}}}}\times 100$$

All the impedance parameters are mentioned in Table 6. The Nyquist plots show capacitive loop depressed at center. The semicircle obtained are not perfect in shape. The deviation of the capacitive semicircles from an ideal semicircle was due to surface roughness and inhomogeneity. This can be overcome by introducing a CPE (constant phase element) to replace the C_dl_ to get more accurate fitting.

10 $${{\rm{Z}}}_{{\rm{CPE}}}=\frac{1}{{{\rm{Y}}}_{o}{({\rm{j}}{\rm{\omega }})}^{{\rm{n}}}}$$

where, Y_0_ = proportional factor, j = imaginary unit (value is equal to √ −1), ω = angular frequency in rad s^−1^ (ω = 2Πf_max_) and n = phase shift and tells the extent of deviation from ideal behavior. When n = −1, the CPE represents an inductor, for n = 0, a pure resistor, and for n = 1, a pure capacitor.

**Table 6:** Electrochemical parameters of impedance for mild steel in 1 M HCl without and with different concentrations of Cys/Ag-Au NCz at 303 K.

Inhibitor concentration (ppm)	R_s_ (Ω cm^2^)	R_ct_ (Ω cm^2^)	CPE		C_dl_ (μFcm^−2^)	χ^2^	% I.E
			Y_0_ × 10^−5^ (Ω^−1^s^n^cm^−2^)	n			
0	0.85	14.68	6.45	0.99782	63.5	0.0696	
30	1.29	31.95	6.34	0.9972	62.3	0.0196	54.07
50	1.97	44.53	5.66	0.9978	55.8	0.0189	67.03
100	2.09	61.87	5.01	0.99809	49.5	0.0230	76.28
150	2.22	117.30	3.99	0.99605	39.2	0.0201	87.49
200	3.53	223.49	2.98	0.99597	29.3	0.0277	93.43
300	2.58	368.17	2.26	0.99602	22.3	0.0301	96.01

The C_dl_ can be calculated by the following equation:

11 $${{\rm{C}}}_{{\rm{dl}}}={{\rm{Y}}}_{o}{({{\rm{\omega }}}_{{\rm{\max }}})}^{{\rm{n}}-1}$$

where ω_max_ = 2πf_max_ (f_max_ = maximum frequency of imaginary component of impedance). The value of R_ct_ increases as the concentration of inhibitor increases which is due to the increase in surface coverage by adsorbed inhibitor molecules on metal-solution interface.

It can be clearly seen from Table 6 that the value of C_dl_ decreases and R_ct_ increases can be explained by Helmholtz model as:

12 $${{\rm{C}}}_{{\rm{dl}}}=\frac{{{\rm{\varepsilon }}}^{0}{\rm{\varepsilon }}}{{\rm{d}}}{\rm{S}}$$

where d is the thickness of electric double layer, S is the surface area of specimen exposed in test medium, ɛ^0^ and ɛ is the permittivity of the air and local dielectric constant of protective layer, respectively. The decrease in the C_dl_ was due to adsorption of inhibitor molecules, which reduces the exposed surface area of electrode in test solution and thus retards the corrosion of mild steel^22^. Furthermore, the water molecules present on the mild steel surface are gradually replaced by inhibitor molecules, which tends to lower the local dielectric constant and thicker the electric double layer^23^.

There is appreciable increase in the diameter of capacitive loops on increasing the inhibitor concentration suggesting the enhancement in the protection at higher concentration. Generally, a high value of R_s_ is obtained in presence of inhibitor as compared to blank due to the reduction in the solution conductivity on adding inhibitor. But in present case the R_s_ is showing an increasing trend upto 200 ppm and then it decreases at 300 ppm so it is not showing any regular trend. This is attributed to the fact that area through which current is passing may not be the same. Bode plots discloses that the process of corrosion is happening through single step with one time constant. It is observed from the bode modulus diagram that the impedance modulus increases with increasing concentration of inhibitor indicating reduction in rate of corrosion in inhibited medium. On increasing the concentration of inhibitor phase angle is approaching to −90°. This indicates that inhibitor does not behave ideally but it approaches towards ideal behavior as some defects and deviation may be present in its route^24^.

### Absorptive film analysis

#### Surface studies

**Figure 11:**
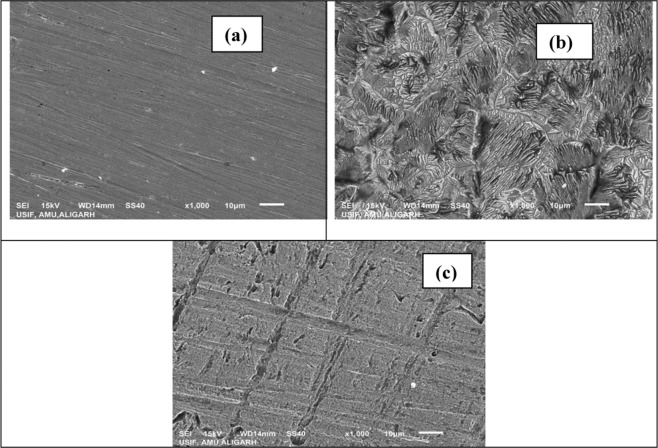
SEM images for mild steel (**a**) polished state (**b**) exposed to 1 M HCl solution (**c**) exposed to 1 M HCl solution containing 300 ppm of Cysteine/Ag-Au nanocomposite for 6 h at 303 K.

The SEM micrographs of polished, uninhibited and inhibited mild steel coupons are shown in Fig. 11. Polished coupon shows defect free smooth surface morphology. Coupon dipped in 1 M HCl for 6 h without inhibitor (blank) shows severely corroded, rough surface with visible pits, cracks and gullies throughout the surface. Mild steel coupon dipped in optimum concentration of inhibitor for 6 h duration shows smooth surface as compared to uninhibited medium with scattered pits on its surface. This observation is suggestive of the formation of protective film on mild steel surface which protected the steel from the attack of aggressive acid.

EDAX had simultaneously been conducted to get the elements available on surface of mild steel before and after commencement of corrosion inhibition experiment. EDAX spectra of polished coupon, uninhibited coupon and coupon immersed in inhibited medium are shown in Fig. S7. EDAX spectra of polished coupon before immersion shows peaks of elements present in composition of steel (C, Mn, Al, V, Fe, Mo). EDAX spectrum of mild steel immersed in 1 M HCl for 6 h shows additional peak of Cl along with suppression in the intensity of Fe peaks. This is attributed to the unhindered HCl corrosion and consequent dissolution of mild steel in acid medium. EDAX spectrum of mild steel in optimum concentration of inhibitor for 6 h shows characteristic peaks of S, N, O, Ag, Au, C and Fe. The presence of these peaks shows that inhibitor is absorbed on mild steel surface through hetero atoms forming a protective layer.

#### FTIR analysis

The adsorption of of Cys/Ag-Au NCz on mild steel surface and involvement of functional groups during adsorption process was predicted by comparing the FTIR spectrum of pure Cys/Ag-Au NCz with that of the absorptive film scrapped from the mild steel surface (Fig. S8). The peaks present in free Cys/Ag-Au NCz were also observed to be present in the scrapped sample with reduction in the intensity of peaks as well as shifting to higher or lower wave numbers. Slight shift in peaks were observed which confirms the interaction of Cys/Ag-Au NCz to metal surface. The shifting of characteristic peaks suggest that present functional groups are involved and are main centre of adsorption process.

## Conclusion

1. The Cys/Ag-Au NCz act as stable and potential green inhibitor against mild steel corrosion in acidic (1 M HCl) medium and the process of corrosion is dependent on concentration, temperature and time.
2. Potentiodynamic polarization data reveals that the behavior of inhibitor was mixed type i.e., it is affecting both anodic process (metal dissolution) as well as cathodic process (hydrogen evolution).
3. EIS result shows that the protective film was formed by the active groups of Cys/Ag-Au NCz on mild steel surface.
4. The negative value of ΔG_ads_ suggest that process of inhibitor adsorption was spontaneous and follows Langmuir adsorption isotherm.
5. The value of Ea and ΔH* increases on increasing inhibitor concentration suggesting that energy barrier for corrosion process to occur increase in presence of Cys/Ag-Au NCz.
6. SEM images shows a less rough surface as compared to uninhibited medium suggesting the formation of protective film in presence of Cys/Ag-Au NCz.

## Supplementary information

Supplementary.
