# Supplementary material for: Cysteine-silver-gold Nanocomposite as potential stable green corrosion inhibitor for mild steel under acidic condition
Source: Sci Rep. 2020 Jan 14;10:279. doi: 10.1038/s41598-019-57181-5 (PMC6959314; doi:10.1038/s41598-019-57181-5)
Supplement: Supplementary file 1 — Supplementary. [file 41598_2019_57181_MOESM1_ESM.docx]

**Cysteine-silver-gold Nanocomposite as potential stable green corrosion inhibitor for mild steel under acidic condition**

**Megha Basik, Mohammad Mobin & Mohd Shoeb**

Corrosion Research Laboratory, Department of Applied Chemistry, Faculty of Engineering and Technology, Aligarh Muslim University, Aligarh 202002, India. Correspondence and requests for materials should be addressed to Mohammad Mobin (email: [drmmobin@hotmail.com](mailto:drmmobin@hotmail.com)).

**Supplementary Information**

**Figure S1.** UV–vis curves of Cysteine/Ag-Au nanocomposite.

**Figure S2.** Log CR vs 1/T plots in absence and presence of different concentration of Cysteine/Ag-Au nanocomposite

**Figure S3.** Log CR/T vs 1/T plots in absence and presence of different concentration of Cysteine/Ag-Au nanocomposite.

**Figure S4.** Langmuir adsorption isotherm graphs of C/θ versus C of WL data at 303-333 K for mild steel in 1 M HCl solution containing different concentration of Cysteine/Ag-Au nanocomposite.

**Figure S5.** Plot of ln K_ads_ versus 1/T for mild steel in 1 M HCl solution containing different concentration of Cysteine/Ag-Au nanocomposite.


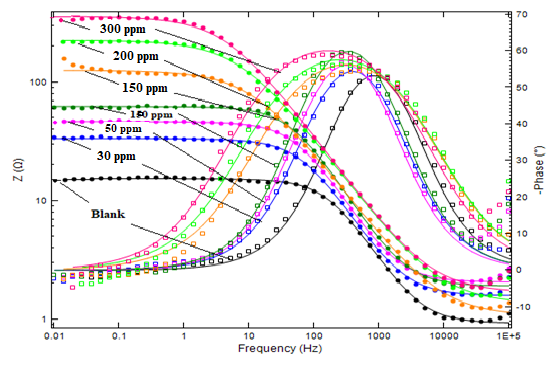


**Figure S6.** Bode modulus and bode phase of Cysteine/Ag-Au nanocomposite at 303 K.

| 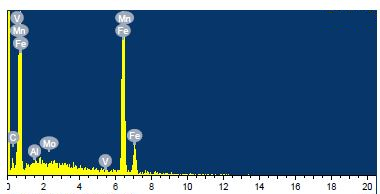 | 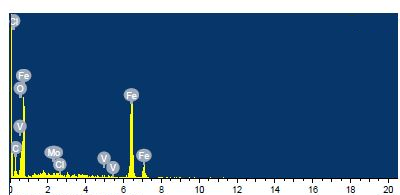 |
| --- | --- |
| 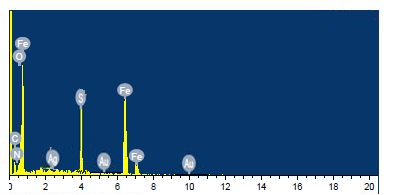 | |

**Figure S7.** EDAX spectra for mild steel (a) polished state, (b) exposed to 1 M HCl solution and (c) exposed to 1 M HCl solution containing 300 ppm Cysteine/Ag-Au nanocomposite at 303 K.

**Figure S8.** FTIR spectra of Cysteine/Ag-Au nanocomposite and Cysteine/Ag-Au nanocomposite adsorbed on mild steel in 1 M HCl.
